# 5-ALA-guided tumor resection during awake speech mapping in gliomas located in eloquent speech areas: Single-center experience

**DOI:** 10.3389/fonc.2022.940951

**Published:** 2022-09-23

**Authors:** Sergey A. Goryaynov, Svetlana B. Buklina, Ivan V. Khapov, Artyom I. Batalov, Alexander A. Potapov, Igor N. Pronin, Artem U. Belyaev, Andrey A. Aristov, Vadim U. Zhukov, Galina V. Pavlova, Evgenii Belykh

**Affiliations:** ^1^ Departments of Neurotraumatology and Neurooncology, N.N.Burdenko National Medical Research Center of Neurosurgery, Moscow, Russia; ^2^ I.M. Sechenov First Moscow State Medical University (Sechenov University), Moscow, Russia; ^3^ Department of Neurogenetics, Institute of Higher Nervous Activity and Neurophysiology, Russian Academy of Sciences, Moscow, Russia; ^4^ Department of Neurosurgery, New Jersey Medical School, Rutgers University, New Jersey, NJ, United States

**Keywords:** glioma, superior longitudinal fasciculus, arcuate fasciculus, fiber tracts, awake craniotomy, fluorescence, 5-ALA

## Abstract

**Background:**

Achieving maximal functionally safe resection of gliomas located within the eloquent speech areas is challenging, and there is a lack of literature on the combined use of 5-aminolevulinic acid (5-ALA) guidance and awake craniotomy.

**Objective:**

The aim of this study was to describe our experience with the simultaneous use of 5-ALA fluorescence and awake speech mapping in patients with left frontal gliomas located within the vicinity of eloquent speech areas.

**Materials and methods:**

A prospectively collected database of patients was reviewed. 5-ALA was administered at a dose of 20 mg/kg 2 h prior to operation, and an operating microscope in BLUE400 mode was used to visualize fluorescence. All patients underwent surgery using the “asleep–awake–asleep” protocol with monopolar and bipolar electrical stimulation to identify the proximity of eloquent cortex and white matter tracts and to guide safe limits of resection along with fluorescence guidance. Speech function was assessed by a trained neuropsychologist before, during, and after surgery.

**Results:**

In 28 patients operated with cortical mapping and 5-ALA guidance (12 Grade 4, 6 Grade 3, and 10 Grade 2 gliomas), Broca’s area was identified in 23 cases and Wernicke’s area was identified in 5 cases. Fluorescence was present in 14 cases. Six tumors had residual fluorescence due to the positive speech mapping in the tumor bed. Transient aphasia developed in 14 patients, and permanent aphasia developed in 4 patients. In 6 patients operated with cortical and subcortical speech mapping and 5-ALA guidance (4 Grade 4, 1 Grade 3, and 1 Grade 2 gliomas), cortical speech areas were mapped in 5 patients and subcortical tracts were encountered in all cases. In all cases, resection was stopped despite the presence of residual fluorescence due to speech mapping findings. Transient aphasia developed in 6 patients and permanent aphasia developed in 4 patients. In patients with Grade 2–3 gliomas, targeted biopsy of focal fluorescence areas led to upgrading the grade and thus more accurate diagnosis.

**Conclusion:**

5-ALA guidance during awake speech mapping is useful in augmenting the extent of resection for infiltrative high-grade gliomas and identifying foci of anaplasia in non-enhancing gliomas, while maintaining safe limits of functional resection based on speech mapping. Positive 5-ALA fluorescence in diffuse Grade 2 gliomas may be predictive of a more aggressive disease course.

## Introduction

Surgery for brain gliomas is aimed at maximizing the extent of resection (EOR) and preserving the quality of life. Improved EOR correlates with recurrence-free survival and overall survival (OS) in glioblastoma (1) and generates interest in the development of techniques to improve EOR such as intraoperative fluorescence guidance (2), awake craniotomy (3), intraoperative MRI (4), and intraoperative ultrasound (5). De Witt Hamer et al. conducted a meta-analysis of 8,091 patients with infiltrative gliomas and demonstrated fewer late neurologic deficits and improved EOR with the use of intraoperative stimulation mapping (6).

However, complete glioma resection is complicated, if not elusive. The infiltrative nature of glioma makes it difficult to define the margin, especially when using operating microscope in a “white-light” mode. To address this issue, several fluorescence-guided surgery (FGS) techniques have been developed; however their effectiveness is mainly limited to high-grade gliomas. In particular, 5-aminolevulinic acid (5-ALA)-based fluorescence guidance improves intraoperative identification of high-grade glioma (HGG) boundaries and therefore increases EOR (7) and results in improved survival rates (8, 9).

The application of 5-ALA-based metabolic navigation is being expanded beyond the surgery of glioblastomas to surgery of low-grade gliomas and non-glial brain tumors (10). At the same time, a controversy remains regarding the added benefit of fluorescence navigation with simultaneous use of direct electrical stimulation and awake mapping in gliomas of eloquent areas (11). Earlier, a number of papers reported on the combined use of 5-ALA and direct electrical stimulation during motor cortex and pyramidal tract mapping (12–14). However, there are very few reports describing the combined use of 5-ALA-based metabolic navigation and awake mapping of the speech areas (15–18). Despite the fact that gliomas are the most common primary brain tumors in adults, only a few emerge near the speech areas and are amenable for surgical resection. Furthermore, advanced techniques such as awake speech mapping and fluorescence navigation are only available in specialized centers, which make such cases unique.

The goal of this paper is to present a single-center experience with combined use of 5-ALA and awake speech mapping for gliomas located in the vicinity of eloquent speech areas focusing on the practical aspects of their combined application. We demonstrate the benefits and limitations of the application of these novel neurosurgical adjuncts, adding to the limited amount of literature available on this topic.

## Materials and methods

### Study design

This study was approved by the local ethics committee. A prospectively collected institutional database of operated patients with gliomas was reviewed and patients of interest were identified. Inclusion criteria were as follows (1): patients age over 18 years old, and (2) diagnosis of left frontal or temporal glioma requiring awake surgery with intraoperative detection of the eloquent speech cortical areas and tracts, including superior longitudinal fasciculus (SLF), arcuate fasciculus (AF), or frontal aslant tract (FAT). We then identified patients with whom 5-ALA-based fluorescence was used for intraoperative guidance.

Patient records were reviewed to identify and summarize information on the combined use of 5-ALA-based navigation and awake speech mapping to define advantages, limitations, and peculiarities for efficient clinical application.

### Imaging

In all cases, preoperative MRI was performed, including gadolinium contrast-enhanced imaging and diffusion-weighted imaging with long-associative tract reconstruction by high angular resolution (3.0 T MRI scanner General Electric Signa HD with 8-channel head coil) to determine if cortical tracts were infiltrated by the tumor. Diffusion tensor imaging protocol included TR = 15,000 ms, TE = minimum, matrix 96 × 96, and FOV = 24 cm, and contained one scan series with b = 1000 and 60 diffusion gradient directions. Processing was carried out using Advantage workstation and READY View software (both from GE Healthcare, Waukesha, WI, USA). Preoperative functional MRI was performed in 20 cases. EOR was defined based on the assessment of pre- and postoperative T1 contrast-enhanced sequences for glioblastomas or T2 FLAIR data for non-contrast-enhancing gliomas. Image assessment was performed by an independent radiologist. Preoperative PET of the brain was not a routine procedure for patients with brain gliomas and was performed only in eight patients.

### Fluorescence guidance

5-ALA (Alasens, SSC NIOPIK, Russian Federation) was administered at a dose of 20 mg/kg 2 h prior to surgery. An operating microscope, Pentero 900 (Carl Zeiss Meditec AG, Obrekochen, Germany), equipped with a fluorescent BLUE400 light module was used to assess and visualize fluorescence.

### Direct cortical and subcortical stimulation

All patients underwent surgery using the “asleep–awake–asleep” protocol (19–21). Transcranial motor evoked potentials were used for neuromonitoring of motor pathways in addition to speech mapping. Cortical and subcortical stimulation was used to determine the location of the eloquent cortical areas and white matter tracts and to delineate the acceptable EOR. After opening the dura mater, direct cortical stimulation was performed with a bipolar 2-pin electrode using rectangular electrical impulses of 50–60 Hz frequency and 3–4 mA amplitude (13). As soon as stimulation-induced speech alterations were identified by a neuropsychological testing, the cortical zones with most apparent and persistent speech disorders were marked by a surgeon. To reduce the risk of intraoperative seizures, continuous electrocorticography was performed during direct electrical stimulation and tumor removal. Stimulation of the white matter tracts was performed using a monopolar loop electrode mounted on a tip of standard vacuum aspirator during the main stage of tumor resection simultaneously with continuous neuropsychological testing. If tumor manipulations caused development or aggravation of speech alterations, resection in this region was stopped. We have previously reported a detailed description of the techniques used for mapping of long association fibers in the glioma surgery elsewhere (21).

### Neuropsychological testing

Hand preference was determined by the Annett Hand Preference Questionnaire (22). The dominant hemisphere for speech was defined by dichotic auditory training with the corresponding coefficient calculation (23).

Speech function was assessed by a trained neuropsychologist before, during, and after surgery. Preoperatively and at discharge, complex neuropsychological testing was performed using the approach developed by Alexander Luria (24). This testing assesses spontaneous speech, word naming, comprehension, repetition, and dictation. Vocabulary was also assessed with a 1-min speech fluency test.

A computerized naming test was used for intraoperative monitoring (25). Patients were asked to name nouns or verbs presented as simple black and white pictures (30 items picturing actions or objects). Also, automatized speech functions were assessed (numbers from 1 to 10, numeration of months and days of week). During this stage of surgery when the cortical stimulation was not used, the patient was kept awake maintaining continuous conversation between the neuropsychologist and the patient. After the completion of tumor resection, the patients were put asleep for the craniotomy closure.

## Results

### Patient population

A prospective single-center database of patients with brain and spinal cord tumors operated with 5-ALA-based metabolic guidance includes 320 patients with gliomas operated from 2008 to 2020. Of those, awake craniotomies were performed in 34 cases. Cortical speech mapping was utilized earlier in our series (*n* = 28), and then, since 2016, we have started using subcortical mapping during awake craniotomies (*n* = 6). Neuropsychological aspects of awake subcortical speech mapping in the latter cohort have been reported previously (21), so here we report results of 5-ALA use with awake speech mapping.

### Surgical protocol

Within the study period, the surgical workflow of combined use of 5-ALA fluorescence and awake speech mapping was optimized as follows. After performing the craniotomy, the patient was awakened and cortical surface was evaluated under the BLUE400 mode of the surgical microscope for possible fluorescent positive areas. Then, we performed direct cortical stimulation using a bipolar probe to map cortical speech positive areas. If fluorescence was identified in the nonfunctional area, then a target biopsy was performed from that area for frozen section analysis. Next, the region for corticotomy was selected outside the eloquent speech areas. Resection then proceeded with an aid of fluorescence guidance. During the tumor resection, dynamic subcortical monopolar stimulation was performed under white light visualization, alternating with fluorescence mode to check for any fluorescent positive areas. We have used a custom monopolar suction device connected to a monopolar stimulation probe to improve resection efficiency similarly to a previous report (26). If speech alterations were encountered, resection was stopped and bipolar electrical stimulation was performed in that area to confirm positive findings. Tumor cavity was then checked again under the blue light for any residual fluorescence.

### Intraoperative findings and outcomes

The overall fluorescence picture in both subgroups (40 patients) looks like that: In Grade IV (glioblastomas), fluorescence was noted in 14 of 16 patients (87,5%); in anaplastic gliomas, fluorescence was revealed in two of seven patients, with five patients being fluorescence-negative; in Grade II gliomas, fluorescence was marked in 4 cases and was absent in 11 cases; in six patients with Grade II–III gliomas, fluorescence was not used; however, intraoperative cortical and subcortical mapping was used in “awake” craniotomy. Thus, glioblastomas exhibited fluorescence considerably and evidently more frequently compared to Grade II–III gliomas.

In the early cohort of 28 patients operated with cortical mapping only, Broca’s area was identified in 23 cases and Wernicke’s area was identified in 5 cases. Among those 28 cases, 12 (43%) were Grade 4, 6 (21%) were Grade 3, and 10 (36%) were Grade 2 (**Table 1**). Fluorescence was present in 14/28 (50%) cases. Among cases with positive fluorescence, majority were Grade 4 glioblastomas (*n* = 10) although 2 glioblastomas were nonfluorescent. At the end of resection under the white light, residual fluorescence was evident in 10 cases, all glioblastomas. After additional resection within functional limits of awake speech monitoring, six tumors had residual fluorescence and four did not. Postoperative MRI was performed within 48 h in 18/28 patients (64%). The EOR was gross total in 12 patients, subtotal in 3 patients, and partial in the remaining 3 patients. In the postoperative period, 14/28 (50%) cases developed transient aphasia and 4/28 (14%) had permanent aphasia.

**Table 1:** Patients with cortical mapping only (prior to 2016), *n* = 28.

**Mapped area (*n* = 28)**	
Broca Wernicke	23/28 (82%) 5/28 (18%)
**Fluorescence pattern/Tumor grade (*n* = 28)**	
Bright Grade 4 Weak Grade 3 Grade 2 Absent Grade 4 Grade 3 Grade 2	10/28 (36%) 10/10 (100%) 4/28 (14%) 1/4 (25%) 3/4 (75%) 14/28 (50%) 2/14 (14%) 5/14 (36%) 7/14 (50%)
**Residual fluorescence after resection under white light (*n* = 14 tumors with fluorescence)**	
Present (all Grade 4) Absent (Grades 2 and 3)	10/14 (71%) 4/14 (29%)
**Residual fluorescence at the end of the surgery (*n* = 10 tumors with residual fluorescence after resection under white light)**	
Present Absent	6/10 (60%) 4/10 (40%)
**Postoperative deficits**	
Transient aphasia Permanent aphasia (at 3 months) Hematoma requiring reoperation	14/28 (50%) 4/28 (14%) 1/28 (3%)

In the later cohort of 12 patients operated with cortical and subcortical speech mapping, cortical speech areas were mapped in 5 cases and subcortical tracts were encountered in all cases (**Table 2**). 5-ALA was used in six cases (*n* = 4 Grade 4 and *n* = 1 Grade 2 gliomas; *n* = 1 anaplastic astrocytoma Grade 3). Additional resection as guided by pink-red fluorescence was possible in five out of six cases to maximize resection extent after resection under white light with dynamic stimulation. However, in each of these cases, positive residual fluorescence was noted at the end of resection because functional limits of resection were reached. In the early postoperative period, 11/12 (92%) cases developed transient aphasia and 1/12 (8%) had permanent aphasia. Postoperative MRI showed gross total resection in 7/12 (58%)—6/7 were operated with 5 ALA; subtotal in 2/12 cases (16%), partial in 2/12 cases (17%), and open biopsy in 1/12 cases (8%).

**Table 2:** Patients with cortical and subcortical mapping (after 2016), *n* = 12.

**Mapped tracts (total *n* = 12)**	
Arcuate fasciculus, superior longitudinal fasciculus Frontal aslant tract	11/12 (82%) 1/12 (18%)
**Fluorescence/Tumor grade (total *n* = 6)**	
Bright Grade 4 Grade 3 Grade 2 5-ALA Not used Grade 2 Grade 3	6/12 (50%) 4/6 (67%) 1/6 (17%) 1/6 (17%) 6/12 (50%) 4 2
**Residual fluorescence after resection under white light (total *n* = 6)**	
Preset (all Grade 4) Absent (Grade 2)	4/6 (83%) 1/6 (17%)
**Residual fluorescence at the end of the surgery (total *n* = 5)**	
Present Absent	5/5 (100%) 0
**New speech deficit during awake craniotomy**	
During monopolar subcortical stimulation while testing the tumor During bipolar subcortical stimulation	6/12 (50%) 6/12 (50%)
**Postoperative deficits**	
Transient aphasia Permanent aphasia Hemiparesis	10/12 (83%) 1/12 (8%) 1/12 (8%)

We did not encounter intraoperative seizures in any of the cases. There were no adverse reactions including skin phototoxicity associated with 5-ALA administration in the studied patients.

### Illustrative cases

For the technique description, we selected five clinical cases illustrating the combined use of metabolic navigation and awake craniotomy.

#### Case 1

**Figure 1:**
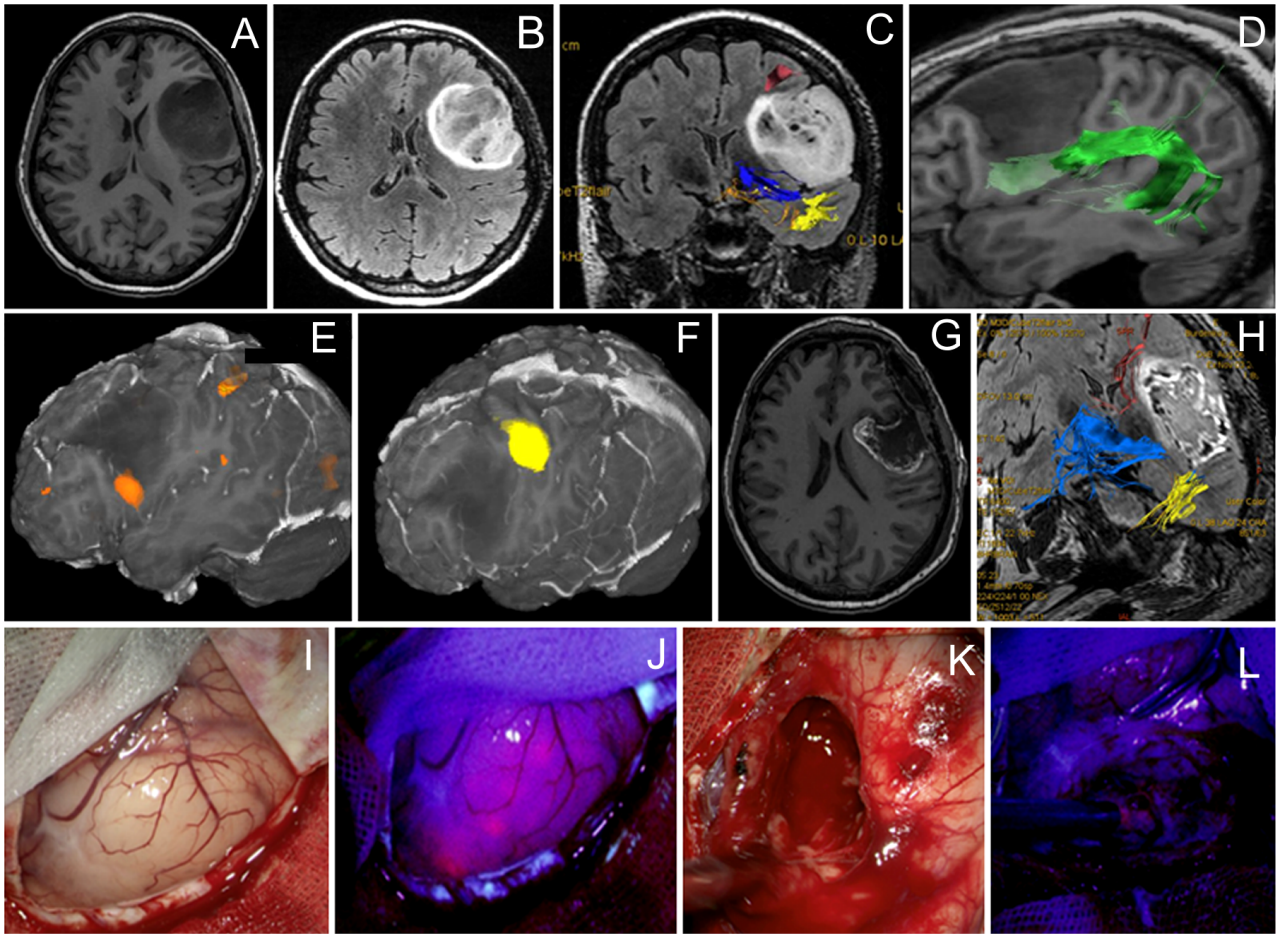
Case 1. A 40-year-old man with the left frontal anaplastic astrocytoma with mild residual fluorescence at the end of resection. Preoperative axial T1-weighted **(A)** and Т2 FLAIR **(B)** MR images demonstrate a clear tumor border. Functional MR images with reconstructed tracts show proximity of **(C)** the long-associative tracts (red—SLF I; blue—IFOF; yellow—ILF), **(D)** arcuate fasciculus (green), **(E)** Broca’s zone (orange) near the anterior–inferior tumor pole, and **(F)** motor arm cortex. **(G)** T1-weighted contrast-enhanced axial MR image 48 h after surgery demonstrating gross total resection (90%). **(H)** Coronal Т2 FLAIR MR image taken within the first 48 h after surgery shows long-associative tracts (blue—AF; yellow—ILF; orange—SLF). Intraoperative visualization of cortex in white mode **(I)** and BLUE400 mode **(J)** demonstrates light pink fluorescence of the left frontal cortex. Images of tumor cavity under white light **(K)** and BLUE400 **(L)** demonstrate light pink fluorescence of the residual tumor fragment.

A 40-year-old man presented with non-enhancing left frontal lobe glioma found on a workup for seizures. During awake craniotomy, cortical mapping revealed speech alterations during direct electrical stimulation at the inferior frontal and inferior part of the precentral gyrus corresponding to Broca’s area. This area corresponded to preoperative fMRI and was located away from the tumor. Evaluation of the brain surface under BLUE400 light showed a mildly fluorescing cortical area (**Figure 1**). A fluorescent positive region was removed with periodic monopolar stimulation under continuous intraoperative neurophysiological monitoring to map the cortical speech areas and the AF. Speech disturbances occurred during monopolar stimulation at a depth of 3 cm and tumor removal was stopped at that area. This location corresponded well to the AF visualized on MR tractography and a small tumor residual was left. Mild residual fluorescence was noted. Postoperatively, the patient had combined temporal (acoustic–mnestic) and frontal (perseverations) lobe aphasias, which regressed completely after 3 weeks of speech therapy. Postoperative Т2 FLAIR MRI showed 90% resection of the tumor. On pathology evaluation, most of the tumor was consistent with diffuse astrocytoma (Grade 2) with a tendency to Grade 3, IDH-I mutant. The patient received procarbazine, lomustine, and vincristine chemotherapy and 56 Gray of radiotherapy and was alive without recurrence on 2 years follow-up.

#### Case 2

**Figure 2:**
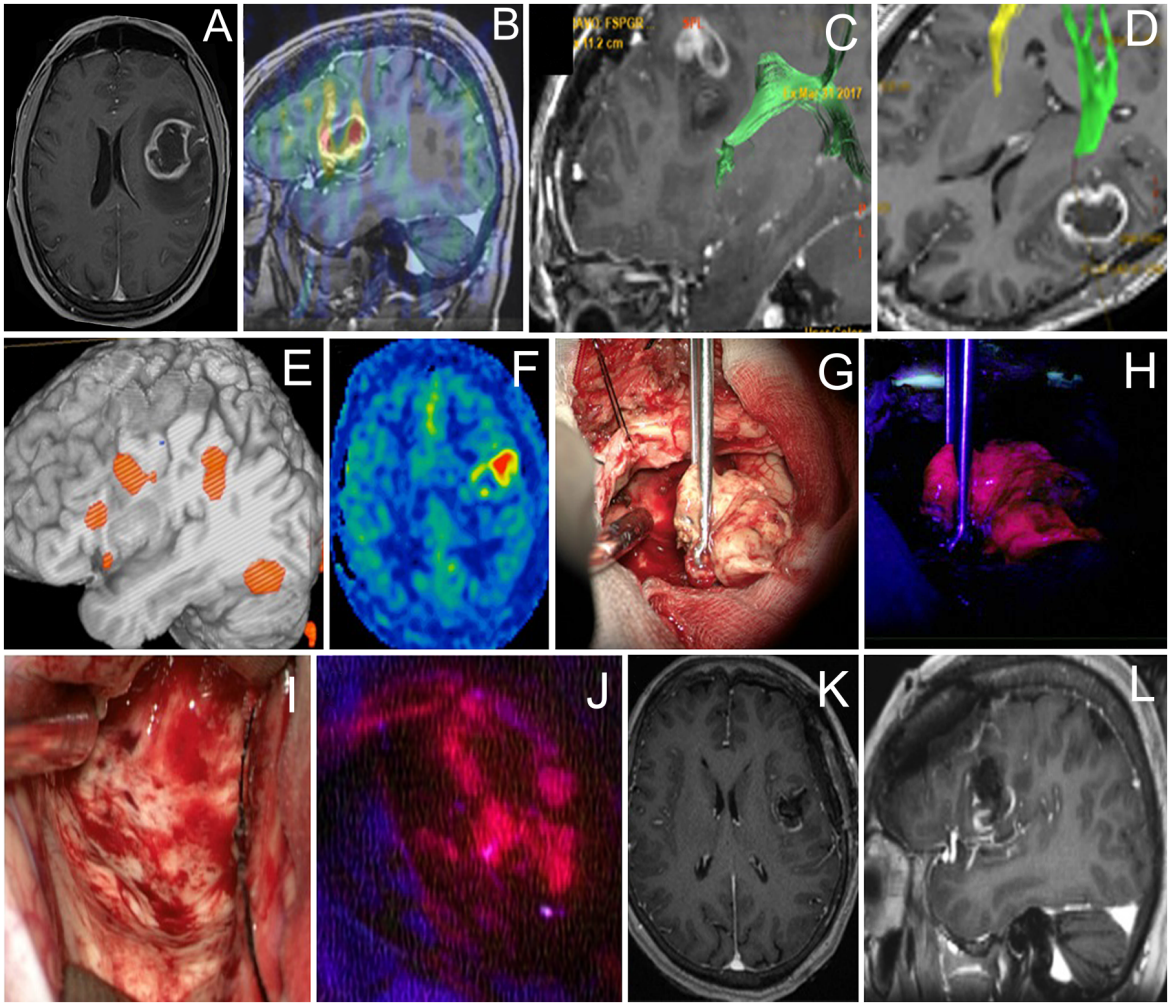
Case 2. A 61-year-old woman with the left frontal glioblastoma located in the Broca’s area astrocytoma with mild residual fluorescence at the end of resection. Axial contrast-enhanced MR image **(A)** and sagittal MR image superimposed on an ASL perfusion map **(B)**, and MR tractography showing proximity of the arcuate fasciculus **(**green in **C)** and pyramidal **(**green in **D)**. Functional MR image demonstrates proximity of the Broca’s area **(E)**. ASL perfusion demonstrates an increased tumor blood flow **(F)**. Intraoperative photo **(G)** of the main tumor bulk that showed bright fluorescence **(H)**. Intraoperative photo of the tumor cavity after tumor resection was stopped due to perseverations developed upon monopolar subcortical stimulation **(I)**. Residual fluorescence is seen in the part of the tumor cavity **(J)**. Postoperative axial **(K)** and sagittal **(L)** contrast-enhanced T1-weighted MR images demonstrate gross total removal of the contrast enhancing tumor part.

A 61-year-old woman with a left frontal glioblastoma presented with mild aphasia. At the beginning of the surgery, cortical stimulation revealed no eloquent speech areas close to the tumor, and the bright tumor fluorescence was seen in BLUE400 mode (**Figure 2**). Resection proceeded with awake neurophysiological speech monitoring and periodic monopolar subcortical stimulation. During subcortical bipolar stimulation at the depth of the surgical cavity, the patient developed combined aphasia and resection was stopped despite a bright residual fluorescence in that area. Postoperative neurological assessment revealed the combination of motor, amnestic, and dynamic aphasias as a result of partial damage of the AF. The patient was given dexamethasone and aphasia regressed completely by postoperative day 21. Postoperative MRI demonstrated subtotal resection. Histological analysis revealed IDH-1 mutant glioblastoma. The patient received 60 Gy radiotherapy and temozolomide, and developed recurrence at 12 months, after which she received three additional cycles of bevacizumab. The patient was alive at 2 years follow-up.

#### Case 3

**Figure 3:**
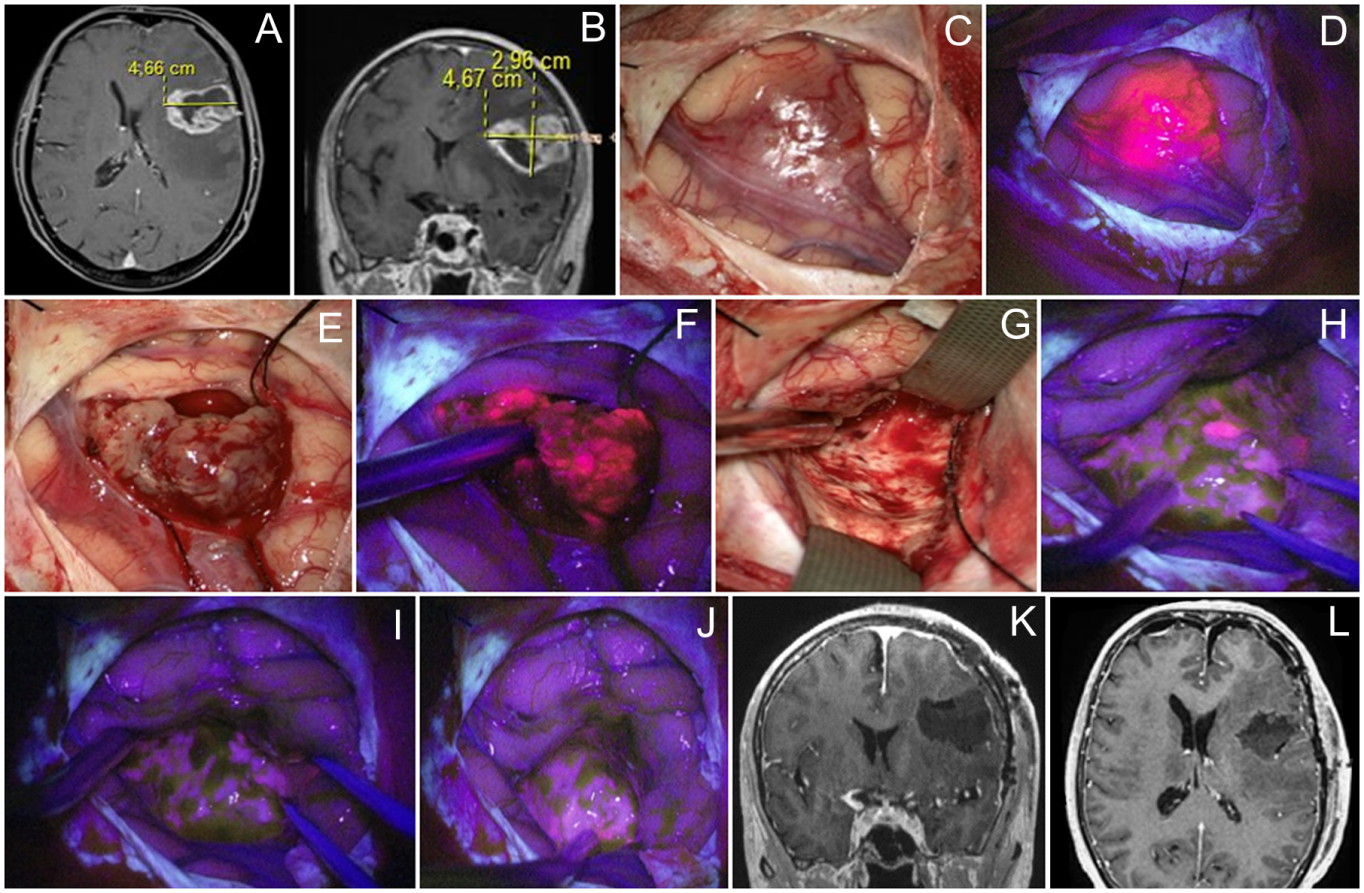
Case 3. A 46 year-old woman with left frontal glioblastoma located in the middle frontal gyrus projection. Contrast-enhanced axial **(A)** and coronal **(B)** MR images. Intraoperative images show that the tumor grows onto the cortex surface **(C)** with bright fluorescence of the cortex in the Blue400 mode **(D)**. Tumor core **(E)** exhibited bright fluorescence **(F)**. At the end of resection under white light **(G)**, residual fluorescent tumor focus is identified and resected after negative subcortical mapping. Final inspection under the Blue400 mode showed no residual fluorescence **(H, I)**; one fluoropositive focus was revealed at the depth of the postoperative cavity **(J)**. Postoperative coronal **(K)** and axial **(L)** contrast-enhanced T1-weighted MR images demonstrate gross total resection of the tumor.

A 46-year-old woman with a left frontal glioblastoma presented with mild motor aphasia. At the beginning of the surgery, cortical stimulation revealed no eloquent speech areas close to the tumor and BLUE400 mode demonstrated a brightly fluorescent tumor in the projection of the middle and inferior frontal gyrus (**Figure 3**). The fluorescent tumor was resected completely and no residual fluorescence was seen at the final stage. The patient remained intact during periodic subcortical stimulation while maintaining dialogue with the surgeon and speech therapist. Postoperative MRI showed gross total resection. In the early postoperative period, neurophysiological assessment revealed mild mixed aphasia that regressed 6 weeks after surgery. Histological analysis revealed IDH 1 mutant glioblastoma. The patient received 60 Gy radiotherapy and temozolomide, and developed recurrence at 10 months, after which she received six cycles of bevacizumab. The patient died 22 months after surgery.

#### Case 4

**Figure 4:**
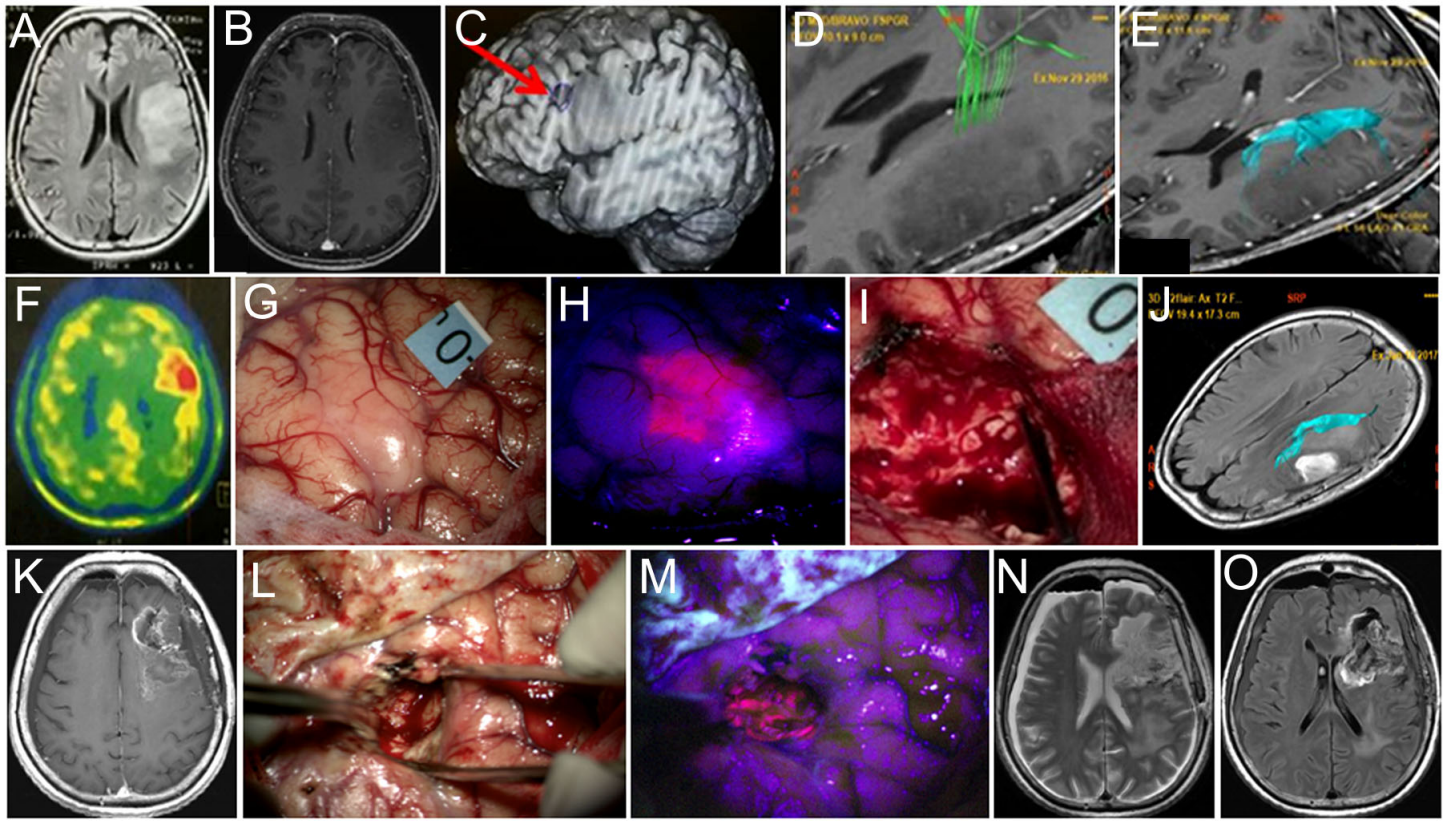
Case 4. A 60-year-old woman with left frontal diffuse astrocytoma that transformed into glioblastoma postoperatively. Preoperative axial Т2 FLAIR **(A)** and contrast-enhanced T1 **(B)** MR images show non-enhancing mass. Broca’s area is indicated by the arrow on the functional MR image **(C)**. MR tractography demonstrates proximity of the pyramidal tract **(D**, green**)** and the arcuate fasciculus **(E**, blue**)**. PET with methionine shows increased drug accumulation (ratio = 1.3) in the tumor **(F)**. Intraoperative image shows right arm motor area labeled with 0 tag **(G)**. Blue400 mode demonstrates bright fluorescence of the invaded cortex **(H)**. Resection was stopped due to speech alteration during bipolar and unipolar stimulation in the deep tumor cavity within the proximity of the AF leaving nonfluorescent tumor residual **(I)**. Postoperative axial Т2-FLAIR MR image with arcuate fasciculus marked in blue demonstrates expected tumor residual **(J)**. Twelve-month follow-up contrast-enhanced T1 MR image demonstrates enhancing tumor focus **(K)**. Intraoperative images during the second surgery in white light **(L)** and Blue400 mode **(M)** demonstrate bright tumor fluorescence. Postoperative axial T2-weighted **(N)** and contrast-enhanced T1 **(O)** MR images demonstrate gross total resection. Images **(A–K)** adapted from (21) courtesy of Goryaynov S.A.

A 60-year-old woman presented with left frontal non-enhancing tumor invading the inferior frontal gyrus found during workup for headache (**Figure 4**). The tumor was located in the proximity of Broca’s area, and long-associated pathways and corticospinal tracts based on fMRI and neuropsychological evaluation revealed minor acoustic–mnestic aphasia. During awake craniotomy, cortical stimulation was performed, and eloquent speech areas to be preserved were labeled. BLUE400 imaging of the brain surface showed brightly pink fluorescence away from Broca’s area. Separate biopsy taken at this area later confirmed infiltration by the tumor cells. This fluorescent part of the tumor was removed completely and the resection proceeded within the subcortical major bulk of the tumor that was not fluorescent. Speech alterations developed during monopolar stimulation at the depth of the resection cavity in the projection of the AF and resection were stopped. Postoperatively, the patient had transient aphasia (a combination of mnestic aphasia, perseverations, and confabulations) that regressed completely by the discharge on postoperative day 10. Postoperative MRI demonstrated expected residual without contrast enhancement and partial tumor removal (70%). Histological analysis showed IDH mutant diffuse astrocytoma Ki 67 < 5% diagnosed as Grade 2 based on the 2016 WHO classification at that time. The patient received radiation therapy (54 Gy) and six cycles of temozolomide. Follow-up MRI after 12 months demonstrated signs of continuous tumor growth with new contrast enhancement, increased perfusion, and increased uptake of methionine on PET, suggesting transformation into anaplastic glioblastoma. The patient was reoperated without speech mapping due to moderate aphasia. During the second surgery, the tumor showed diffuse bright pink fluorescence allowing for gross total resection as confirmed by postoperative contrasted MRI. In the postoperative period, the patient had moderate aphasia and hemiparesis. Histological assessment showed IDH mutant glioblastoma. The patient received 60 Gy radiation therapy, temozolomide, and bevacizumab chemotherapy and was alive at follow-up 28 months after the first surgery.

#### Case 5

**Figure 5:**
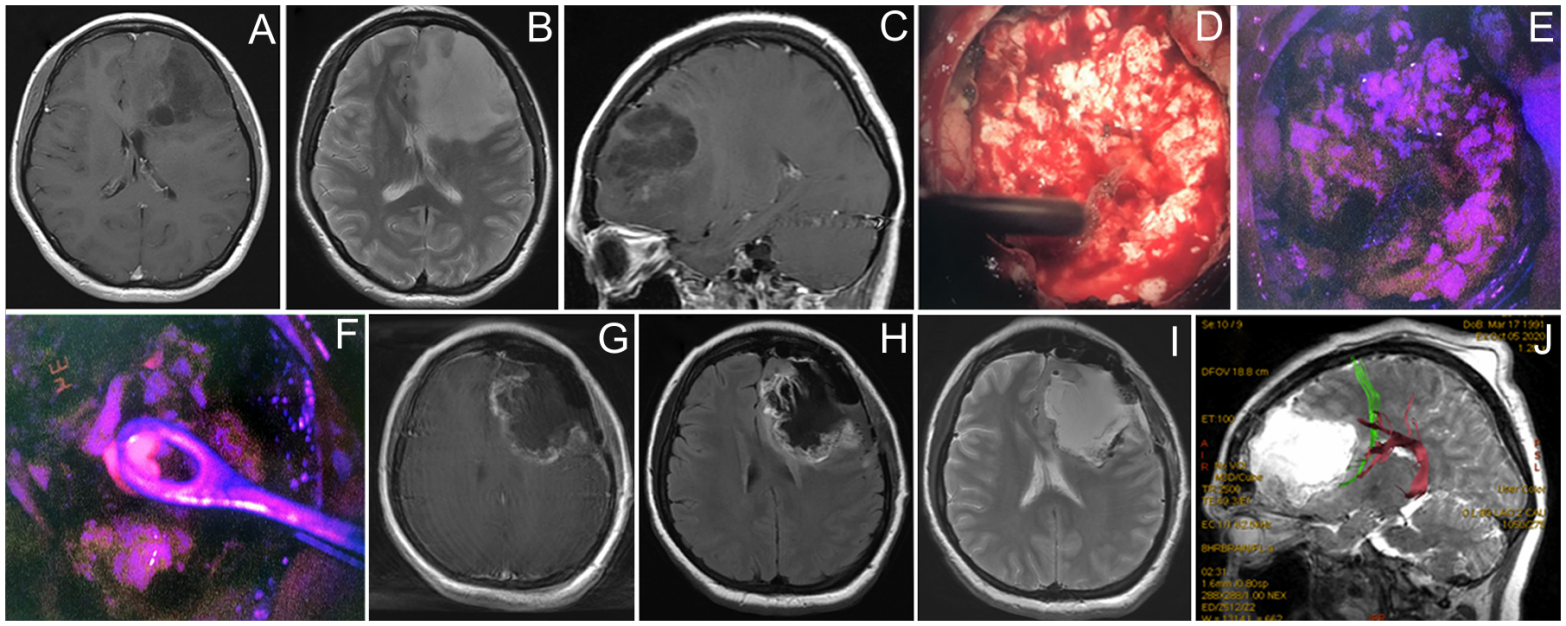
Case 5. A 29-year-old woman with left frontal non-enhancing glioma with a focus of intratumoral fluorescence. Preoperative contrast-enhanced T1 axial **(A)**, T2 axial **(B)**, and contrast-enhanced T1 sagittal MR images showing the tumor **(C)**. Intraoperative photos of the tumor bulk in the white mode **(D)** and fluorescence mode **(E)** image demonstrate dim pink fluorescence. A spot of bright fluorescent tissue was identified **(F)** and taken for biopsy. Postoperative T1 contrast-enhanced **(G)**, T1 **(H)**, and T2 **(I)** MR images demonstrate partial tumor resection. Postoperative MR tractography overlaid on a sagittal MR T2 image demonstrates frontal аslant tract (green) and arcuate fasciculus (purple) close to the posterior margin of postoperative cavity **(J)**.

A 29-year-old woman **Figure 5** presented with a single episode of generalized seizures and was found to have a large non-enhancing left frontal tumor on the MRI. Awake speech mapping was performed in a standard fashion. Cortex and main bulk of the tumor showed no visible fluorescence. However, in the midst of the tumor, a focus of moderate fluorescent positive tissue was found. The remaining tumor showed no visible fluorescence and resection continued with an ultrasound aspirator under continuous dynamic control of subcortical monopolar stimulation. Speech alterations were detected upon subcortical stimulation near the posterior tumor pole in the proximity of AF and FAT; thus, the resection was limited in that direction. Postoperative MRI demonstrated expected residual and overall partial tumor resection (70%). Postoperatively, the patient remained neurologically intact and was discharged home. Histological assessment of the tumor bulk showed diffuse astrocytoma; however, targeted biopsy of the fluorescent area showed IDH 1 mutant anaplastic astrocytoma, which allowed classifying this tumor as Grade 3. The patient received six cycles of temozolomide and 60 Gy radiation therapy. Tumor recurred 30 months after surgery and chemotherapy was resumed. The patient is alive 3 years after the surgery.

## Discussion

### Benefits of 5-ALA in glioblastoma surgery

Overall, among the all studied patients, 20 had fluorescent tumors. In four cases of the early cohort operated with cortical mapping only, we were able to resect residual 5-ALA fluorescent spots and achieve complete resection without residual fluorescence. In the cohort of six patients operated with subcortical mapping and 5-ALA guidance, we were able to extend resection based on the 5-ALA imaging guidance in five glioblastoma patients. However, in none of these cases was complete resection of fluorescent tissue possible due to speech alterations during continuous awake monitoring. Although some residual fluorescent tissue was deliberately left, maximizing EOR would not be possible without fluorescence guidance in such cases. The resection was stopped due to speech disturbances even in the presence of residual fluorescence in 12/20 (60%) of those cases. Our study was not designed to assess the clinical significance of added benefit of the resection extent in cases where some residual was left due to positive speech mapping. Nevertheless, data from prior studies suggest that such difference is perhaps significant.

Glioma resection can be challenging due to the difficulty in differentiating between the infiltrative tumor and healthy brain tissue under a standard “white-light” visualization. Because of this, many neurosurgeons adopted fluorescence-guidance techniques to improve intraoperative demarcation of tumor boundaries (7, 27, 28). Studies evaluating the efficacy of fluorescence guidance in the surgery of HGG demonstrated that 5-ALA fluorescence boundaries exceeded those of the gadolinium contrast enhancement on MRI (29). Analysis of outcomes of patients operated with 5-ALA-based fluorescence demonstrated an increased gross total resection rate and overall and 6-month recurrence-free survival (8, 9). Whether it is reasonable to perform awake craniotomy in patients with HGG is being discussed in the literature, with some of the authors arguing for its use only for low-grade gliomas because of a considerably lower incidence of aphasia in these patients (30). Current techniques of awake craniotomy allow for radical and safe resection of HGG located in the vicinity of speech networks and may lead to lesser number of postoperative complications and increased survival (31).

According to our data, up to 6% of glioblastomas are fluorescence-negative (10). To synthesize one molecule of protoporphyrin IX (PpIX), eight 5-ALA molecules are needed; separate steps of heme synthesis provoke catalyzation of special enzymes, among which ferrochelatase (or protoporphyrin ferrochelatase) and porphobilinogen deaminase are specially interesting, as their activity is increased in tumor cells compared to normal brain tissues (32). Absence of fluorescence in glioblastoma cases may probably be related to heterogeneity of the tumor and its insufficient haem biosynthesis enzyme activity.

### Experience with 5-ALA during awake craniotomy

Earlier, a number of publications described the combined use of 5-ALA-based fluorescence and direct electrical stimulation for motor cortex and pyramidal tract mapping (13, 14, 33). There are only a few papers reporting on the combined use of 5-ALA and awake mapping of eloquent speech areas and long-associative tracts (15–18). In a series of (34), 2 out of 26 patients underwent awake speech mapping with 5-ALA-based FGS, and in one of them, resection was incomplete owing to identification of eloquent fibers during mapping (17). Corns et al. (15) reported that one case report of a recurrent glioblastoma near Broca’s area completely resected with 5-ALA-based FGS and awake speech mapping (15). Skjøth-Rasmussen et al. (16) assessed 19 patients with HGG in eloquent areas in whom combination of 5-ALA and awake mapping was used and reported complete or near-complete tumor removal in 10 of 19 cases and a GTR of 6 out of 9 patients with newly diagnosed glioblastoma (16). Henderson et al. presented a video of the simultaneous use of a headlamp for visualization of fluorescence during awake craniotomy with bilingual speech mapping for left temporal lobe glioblastoma (18). In the current study, we assessed 34 patients who underwent awake craniotomy for speech mapping and 5-ALA-based fluorescence guidance tumor resection.

We believe that such combination is reasonable for resection of small HGGs located near the AF or cortical language areas. In general, resection of the brightly fluorescent parts of the tumor is considered safe, as it represents a solid part of the tumor, while vague pink fluorescence may indicate tumor infiltration of a surrounding brain that may be functionally eloquent (35). The use of intraoperative stimulation is therefore of utmost importance to identify those eloquent areas that exhibit fluorescence in order to preserve them and prevent neurologic deficit. For this reason, we view these techniques as complementary in the surgery of HGG located in the proximity of eloquent speech networks. As opposed to large high-grade tumor patients who usually have preexistent aphasia and therefore not eligible for awake craniotomy, small HGGs are the best candidates for this combined technique.

The rationale for the application of both techniques in the surgery of low-grade gliomas located in proximity to the eloquent speech areas is different. Awake speech mapping is an established intraoperative technique for such tumors, but the simultaneous use of 5-ALA is debatable, as PpIX fluorescence is not always seen in low-grade tumors. However, we have shown a rather high incidence (up to 40%) of tumor fluorescence in such tumors, which could provide clinically useful information discussed below (36, 37).

### Benefits and limitations of 5-ALA during awake glioma surgery

First, it is possible to perform safe resection of the infiltrative HGGs with visible PpIX fluorescence and preserve eloquent long-associative tracts at the depth of the surgical cavity that may be infiltrated by the tumor cells. Neuromonitoring (including awake speech mapping) in such cases allows one to pause resection in a timely manner, despite the presence of residual fluorescence, and to avoid postoperative deficits (38). In all five patients with HGG, we encountered speech alterations during subcortical stimulation and therefore deliberately left fluorescent-positive tumor areas to avoid the risk of speech deficit (as examples, pale fluorescence in illustrative case 1 and strong fluorescence in case 2). Feigl et al. analyzed a series of gliomas near eloquent areas resected with 5-ALA-based FGS and asleep motor and sensory evoked potential neuromonitoring and demonstrated that resection was stopped in 24% (6 out of 25 cases) despite the remaining fluorescence due to changes found in neuromonitoring; however, they did not use speech mapping (12). It should be noted that even in the presence of some pale residual fluorescence, postoperative MRI may show no residual contrast enhancement, which could be explained by the fact that the zone of 5-ALA fluorescence is larger than the zone of gadolinium contrast enhancement in HGGs (29).

Second, in diffuse low-grade gliomas, visible fluorescence may predict anaplastic transformation. As illustrated in case 2, the encountered fluorescence of the cortex was unusual for diffuse astrocytoma. All fluorescent portions of the tumor were safely removed; however, the resection was stopped near the AF, leaving some of the fluorescence-negative tumor tissue. The tumor recurred in 1 year, this time demonstrating malignization into glioblastoma. Thus, the use of awake craniotomy allowed us to outline the zone of functional boundaries for safe resection, while fluorescence was helpful in predicting prognosis of the disease. Several studies have also indicated that just the mere presence of 5-ALA-based fluorescence in WHO Grade 2 gliomas is a factor correlating with worse progression-free survival, malignant transformation free survival, and OS (39, 40). There is a strong need for further investigation of the prognostic role of 5-ALA-based fluorescence in the surgery of Grade 2 gliomas.

Third, the areas of strong 5-ALA-based fluorescence in non-contrast enhancing Grade 2–3 gliomas represent anaplastic tumor foci (36, 41–43). This allowed us to take samples of the most representative tumor regions for histological analysis, which would otherwise be missed under white light, while resecting the rest of the nonfluorescent tumor parts under functional guidance of monopolar or bipolar stimulation (case 5). 5-ALA-based intraoperative guidance has insufficient sensitivity in the surgery of anaplastic gliomas, up to 67% (10), and Grade 2 gliomas, ranging from 5% to 46% (37). 5-ALA therefore is not used to define the EOR in low-grade tumors, but to identify the most active tumor parts for biopsy acquisition and prognostication. Electrical stimulation of the long-associative pathways (FAT and AF) is especially important for controlling the safety of resection near the tumor margins, both with and without residual fluorescence. It could also be argued that patients may choose to have certain postoperative neurological deficits to allow for extension of the tumor resection to hopefully improve PFS and possibly OS.

Known limitations of the 5-ALA fluorescence technique include added time during the surgery to visualize the fluorescence, and the need for special fluorescent modules for microscope or loupes. The operating room and postoperative area were maintained darkened to prevent phototoxic 5-ALA reactions and did not negatively affect interaction during the awake portion of the craniotomy.

## Conclusion

5-ALA-based metabolic navigation and awake speech mapping are complementary intraoperative adjuncts that could be used simultaneously and interchangeably for the surgery of LGG and HGG located near the eloquent speech areas. This combination allows one to maximize the extent of tumor resection in cases of HGG, perform targeted biopsy of the most aggressive areas, and obtain additional prognostic information based on the presence of intraoperative fluorescence in LGG, and to reduce risks of permanent postoperative speech deficits by identification and preservation of the eloquent cortical areas and white matter associative tracts, even in the presence of fluorescence.

## Data availability statement

The original contributions presented in the study are included in the article/supplementary material. Further inquiries can be directed to the corresponding author.

## Ethics statement

The studies involving human participants were reviewed and approved by Burdenko Neurosurgical Center. The patients/participants provided their written informed consent to participate in this study.

## Author contributions

Supervision: AAP and INP. Patients were under the care of SAG and VUZ. Data acquisition and provision of important intellectual content: SAG, SBB, AIB, AAP, AUB, AAA, VUZ, GVP. The article was drafted by SAG, EB, and IVK. Figures and article revised by SAG, EB, and AAA. Final approval: all authors. All authors contributed to the article and approved the submitted version.

## Funding

This research was funded by the Ministry of Science and Higher Education of the Russian Federation, grant number 075-15-2020-809 (13.1902.21.0030)

## Acknowledgments

Special thanks to Natalia Pestovskaya for translating the paper. Special thanks to Okhlopkov V.A., Kosyrkova A.V., Zakharova N.E., Shugai S.V., and Ogurtsova A.A. Data presented in this work are included in a doctoral dissertation of SG.

## Conflict of interest

The authors declare that the research was conducted in the absence of any commercial or financial relationships that could be construed as a potential conflict of interest.

## Publisher’s note

All claims expressed in this article are solely those of the authors and do not necessarily represent those of their affiliated organizations, or those of the publisher, the editors and the reviewers. Any product that may be evaluated in this article, or claim that may be made by its manufacturer, is not guaranteed or endorsed by the publisher.
